# α-Pyrone Polyketides from *Streptomyces ambofaciens* BI0048, an Endophytic Actinobacterial Strain Isolated from the Red Alga *Laurencia glandulifera*

**DOI:** 10.3390/md15120389

**Published:** 2017-12-14

**Authors:** Enikő Rab, Dimitrios Kekos, Vassilios Roussis, Efstathia Ioannou

**Affiliations:** 1 Section of Pharmacognosy and Chemistry of Natural Products, Department of Pharmacy, School of Health Sciences, National and Kapodistrian University of Athens, Panepistimiopolis Zografou, Athens 15771, Greece; rabeniko@gmail.com (E.R.); roussis@pharm.uoa.gr (V.R.); 2 Laboratory of Biotechnology, School of Chemical Engineering, National Technical University of Athens, 9 Iroon Polytechniou Str., Zografou Campus, Athens 15780, Greece; kekos@chemeng.ntua.gr

**Keywords:** *Streptomyces ambofaciens*, algicolous actinobacterium, endophytic strain, polyketides, α-pyrone, structure elucidation, bioactivity evaluation

## Abstract

Four new (**1**–**4**) and six previously reported (**5**–**10**) α-pyrone polyketides, along with benzoic acid, hydrocinnamic acid, and (*E*)-cinnamic acid, were isolated from the organic extract resulting from the cultivation of the algicolous strain *Streptomyces ambofaciens* BI0048, which in turn was isolated from the inner tissues of the red alga *Laurencia glandulifera*. The structure elucidation of the isolated natural products was based on extensive analysis of their spectroscopic data (NMR, MS, UV, IR). Compounds **1**–**10** were evaluated for their antibacterial and cytotoxic activities against two multidrug-resistant strains of *Staphylococcus aureus* and one strain of *Escherichia coli*, as well as two human cancer cell lines.

## 1. Introduction

The screening of microbial natural products represents an important route to the discovery of novel anticancer and antibiotic agents [1,2,3]. Diverse actinobacteria isolated from unique ecosystems have been shown to produce bioactive compounds, which exert their influence by mechanisms that are not compromised by existing multidrug-resistance pathways [4].

In order to obtain new strains likely to produce novel metabolites, investigation of samples from different habitats and extreme environments is necessary. The East Mediterranean basin is a geomorphologically and biologically unique marine ecosystem that has not been examined so far for its microbiota as producers of secondary metabolites.

In search of new bioactive secondary metabolites from marine organisms of the Greek seas, we have recently expanded our research interests in marine-derived microbiota. To this end, we have selectively isolated more than 900 actinobacterial strains from marine sediments and marine macroorganisms collected from the Aegean and the Ionian Seas.

On the basis of preliminary screening of the chemical profiles of extracts obtained from small-scale liquid cultures of numerous actinobacterial strains with LC-DAD-MS and NMR in conjunction with the results of the evaluation of their antibacterial and cytotoxic activities, strain BI0048, isolated from the inner tissues of the red alga *Laurencia glandulifera*, was selected for further chemical investigation. The algicolous endophytic strain, which was identified as *Streptomyces ambofaciens*, was cultured in large-scale in flasks containing a seawater-based liquid medium and the resulting organic residue derived from its extraction was subjected to a multi-step fractionation scheme that led to the isolation of a number of secondary metabolites.

**Figure 1:**
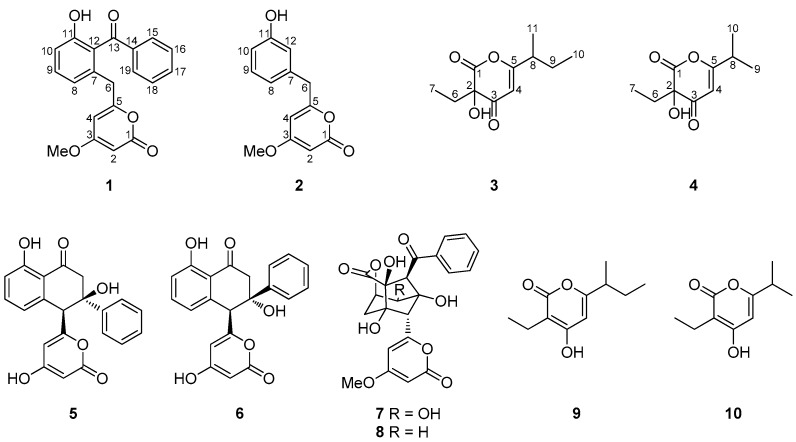
Chemical structures of compounds **1**–**10** isolated from the algicolous endophytic strain *Streptomyces ambofaciens* BI0048.

Herein, we report the isolation and structure elucidation of four new (**1**–**4**) and six previously reported (**5**–**10**) α-pyrone polyketides (Figure 1) and the evaluation of their antibacterial and cytotoxic activities.

## 2. Results and Discussion

The organic extract resulting from the cultivation of the algicolous endophytic strain *S. ambofaciens* BI0048 was subjected to repetitive chromatographic fractionations and HPLC purifications to afford the new α-pyrone polyketides **1**–**4** and nine previously reported metabolites, which were identified as wailupemycin D (**5**), wailupemycin E (**6**), enterocin, also known as vulgamycin (**7**), 5-deoxy-enterocin (**8**), germicidin A (**9**), germicidin B (**10**), benzoic acid, hydrocinnamic acid, and (*E*)-cinnamic acid by comparison of their spectroscopic and physical characteristics with those reported in the literature [5,6,7,8,9,10,11].

**Figure 2:**
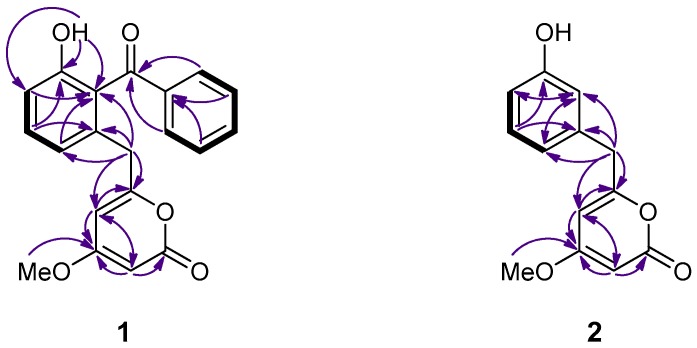
COSY (bold bonds) and important HMBC (arrows) correlations observed for compounds **1** and **2**.

Zoumbericin A (**1**), isolated as a colorless oil, displayed an ion peak at *m*/*z* 335.0928 (HRESIMS), corresponding to C_20_H_15_O_5_ and consistent with [M − H]^−^. The ^13^C NMR spectrum (Table 1) revealed 20 carbon signals, which corresponded to eight non-protonated carbon atoms, among which two carbonyls at δ_C_ 165.2 and 199.2, ten methines, one methylene and one methyl, as determined from DEPT experiments. The ^1^H NMR spectrum (Table 1) included signals at δ_H_ 6.87 (1H, d, 7.5 Hz), 7.36 (1H, dd, 8.2, 7.5 Hz) and 6.95 (1H, d, 8.2 Hz) indicative of a 1,2,3-trisubstituted aromatic ring, as well as at δ_H_ 7.67 (2H, d, 8.3 Hz), 7.43 (2H, dd, 8.3, 7.4 Hz) and 7.57 (1H, t, 7.4 Hz) pointing to the presence of a monosubstituted aromatic ring. Furthermore, the ^1^H NMR spectrum of **1** exhibited signals for a deshielded methylene at δ_H_ 3.52, a methoxy group at δ_H_ 3.69, and two methines at δ_H_ 5.27 and 5.43. In agreement with the literature [8,9,12], the latter three signals, in conjunction with the ^13^C NMR resonances of C-1 (δ_C_ 165.2), C-2 (δ_C_ 87.8), C-3 (δ_C_ 170.9), C-4 (δ_C_ 101.3) and C-5 (δ_C_ 162.8), were characteristic for a 4,6-disubstituted 4-methoxy-α-pyrone ring, present also in the co-occurring enterocin (**7**) and 5-deoxy-enterocin (**8**). The absorption band at 1687 cm^−1^ and the maximum absorbance at 282 nm observed in the IR and UV spectra of **1**, respectively, further supported the presence of the α-pyrone ring in the molecule. The HMBC correlations of H_2_-6 to C-4, C-5, C-7, C-8 and C-12 suggested the linkage of the α-pyrone ring to the 1,2,3-trisubstituted aromatic ring through C-6, while the monosubstituted aromatic ring was linked to the latter through C-13 (Figure 2). This hypothesis was further supported by the fragment ions at *m*/*z* 105 and 259 observed in the EIMS of **1**, corresponding to the phenylketone moiety ([C_7_H_5_O]^+^) and [C_14_H_11_O_5_]^+^ resulting from the cleavage of the phenyl group.

Zoumbericin B (**2**), obtained as a colorless oil, had the molecular formula C_13_H_12_O_4_, as calculated from the HRESIMS measurements and NMR data. The spectroscopic characteristics of **2** were similar to those of metabolite **1**. Specifically, as in the case of **1**, the ^1^H and ^13^C NMR spectroscopic data of compound **2** (Table 1) included signals for a 4,6-disubstituted 4-methoxy-α-pyrone moiety and a deshielded methylene resonating at δ_H_ 3.67 as a singlet. However, in contrast to **1**, the only other signals present in the NMR spectra of **2** were those attributed to a 1,3-disubstituted aromatic ring. The COSY and HMBC correlations observed for **2** (Figure 2) allowed for the unambiguous identification of the 4-methoxy-α-pyrone and the aromatic ring which were connected through C-6.

**Figure 3:**
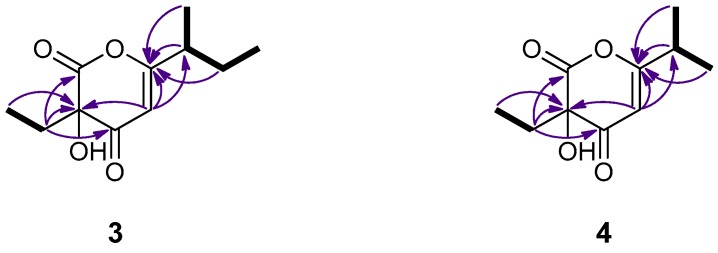
COSY (bold bonds) and important HMBC (arrows) correlations observed for compounds **3** and **4**.

Germicidin K (**3**), isolated as a colorless oil, displayed an ion peak at *m*/*z* 211.0975 (HRESIMS), corresponding to C_11_H_15_O_4_ and consistent with [M − H]^−^. The ^1^H NMR data of **3** (Table 2), including signals for two aliphatic methyls on secondary carbons (δ_H_ 0.92 and 0.97), one aliphatic methyl on a tertiary carbon (δ_H_ 1.20), two methylenes (δ_H_ 1.54/1.68 and 1.98), one relatively deshielded methine (δ_H_ 2.41) and one olefinic methine (δ_H_ 5.68), were rather similar to those of the co-occurring germicidin A (**9**). Indeed, the ^13^C NMR spectrum of **3** exhibited 11 carbon signals, which, according to the DEPT experiments, were attributed to three methyls, two methylenes, two methines, and four quaternary carbon atoms. However, in contrast to germicidin A (**9**), which exhibits signals for one carbonyl (δ_C_ 167.5) and four olefinic carbons (δ_C_ 99.6, 104.7, 165.9 and 167.2) [10], resonances for two carbonyls (δ_C_ 167.2 and 191.2), two olefinic carbons (δ_C_ 104.6 and 175.3) and one oxygenated quaternary carbon (δ_C_ 91.7) were evident for compound **3** (Table 2). The structure of germicidin K (**3**) was proposed on the basis of the correlations observed in its 2D NMR spectra, as depicted in Figure 3. Due to the limited amount in which **3** was isolated, it was not possible to determine the absolute configuration at C-2 and C-8.

Germicidin L (**4**), with the molecular formula C_10_H_14_O_4_, as deduced from the HRESIMS measurements where an ion peak consistent with [M − H]^−^ was observed at *m*/*z* 197.0818, was obtained as a colorless oil. Its ^1^H and ^13^C NMR spectroscopic data (Table 2) closely resembled those of **3** and the co-occurring germicidin B (**10**), with the most prominent differences between **3** and **4** being the presence of a second aliphatic methyl on a tertiary carbon and the simultaneous absence of an aliphatic methyl on a secondary carbon and a methylene. As in the case of **3**, the ^13^C NMR spectrum of **4** included signals for two carbonyls (δ_C_ 167.6 and 191.3), two olefinic carbons (δ_C_ 103.3 and 176.2) and one oxygenated quaternary carbon (δ_C_ 91.7), instead of one carbonyl (δ_C_ 168.2) and four olefinic carbons (δ_C_ 98.6, 104.7, 166.5 and 167.8), as observed for germicidin B (**10**) [10]. The COSY cross-peaks and the HMBC correlations, as depicted in Figure 3, supported the proposed structure of germicidin L (**4**). Similarly, since **4** was isolated in limited amount, the absolute configuration at C-2 could not be established. 

The α-pyrone moiety constitutes an essential pharmacophore in many naturally occurring and synthetic bioactive compounds. Natural products featuring a α-pyrone ring are often involved in defense processes, while frequently they possess antibacterial, antifungal, antiviral, cytotoxic, phytotoxic and neurotoxic properties [13,14]. In 1976, when initially isolated and characterized, enterocin (**7**) was reported to be bacteriostatic in a disk-diffusion assay at a concentration of 4 mg/mL against Gram-positive and Gram-negative bacteria, including strains of *Escherichia coli*, *Proteus vulgaris*, *Sarcina lutea*, *Staphylococcus aureus* and *Corynebacterium xerosis*, but showed no activity against strains of *Bacillus subtilis*, *Bacillus megaterium*, *Pseudomonas aeruginosa*, *Candida albicans* and *Penicillium chrysogenum* [7]. In a subsequent study, enterocin (**7**) did not exhibit any activity against strains of *S. aureus*, *B. subtilis*, *P. aeruginosa*, *Salmonella typhimurium*, *E. coli*, *Saccharomyces cerevisiae*, *C. albicans*, *P. chrysogenum* and *Trichophyton mentagrophytes* and displayed weak activity only against a strain of *Micrococcus luteus* [15]. In addition, enterocin (**7**) exhibited herbicidal activity when applied post-emergence, controlling dicotyledonous weeds and grasses at dosages between 125 and 500 g ha^−1^, being safe for application on cotton, maize and barley [16]. 5-Deoxy-enterocin (**8**) was reported to inhibit strains of *S. lutea*, *S. aureus*, *Klebsiella pneumoniae* and *Vibrio percolans* at a concentration of 0.5 mg/mL [9]. Germicidin A (**9**), the first known autoregulative inhibitor of spore germination in the genus *Streptomyces*, has been shown to have an inhibitory effect on the germination of *Streptomyces* arthrospores at concentrations as low as 40 pg/mL, while at higher concentrations it inhibited porcine Na^+^/K^+^-activated ATPase and retarded the germination of the cress *Lepidium sativum*. In contrast, germicidin B (**10**) did not show any activity in the same germination and ATPase assays [10]. Moreover, germicidin A (**9**) exhibited weak activity against strains of *Streptomyces viridochromogenes* and *Streptomyces griseus*, but did not inhibit the growth of other Gram-positive and Gram-negative bacteria and several fungi and showed no effect on the mobility of the nematode *Caenorabditis elegans* [10]. Furthermore, germicidins A (**9**) and B (**10**) were proven inactive in a disk-diffusion assay against strains of *B. subtilis*, *Mycobacterium vaccae*, *P. aeruginosa*, methicillin-resistant *S. aureus*, vancomycin-resistant *Enterococcus faecalis* and *Sporobolomyces salmonicolor* [17].

Compounds **1**–**10** were evaluated for their antibacterial activities against the epidemic methicillin-resistant strain EMRSA-15 and the multidrug-resistant effluxing strain SA1199B of *S. aureus*, as well as the *E. coli* strain NCTC-10418. Furthermore, the cytotoxic activities of **1**–**10** were tested against the MCF7 (breast adenocarcinoma) and A549 (lung carcinoma) human cancer cell lines. However, metabolites **1**–**10** were proven inactive in both bioactivity assays.

## 3. Materials and Methods

### 3.1. General Experimental Procedures

Optical rotations were measured on a Perkin Elmer model 341 polarimeter (PerkinElmer Instruments, Norwalk, CT, USA) with a 1 dm cell. UV spectra were obtained on a Perkin Elmer Lambda 40 spectrophotometer (PerkinElmer Ltd., Buckinghamshire, UK). IR spectra were obtained on a Bruker Tensor 27 spectrometer (Bruker Optik GmbH, Ettlingen, Germany). NMR spectra were recorded on Bruker AC 200 and Bruker DRX 400 spectrometers (Bruker BioSpin GmbH, Rheinstetten, Germany). Chemical shifts are given on a δ (ppm) scale using TMS as internal standard. The 2D NMR experiments (HSQC, HMBC, COSY) were performed using standard Bruker pulse sequences. High-resolution ESI mass spectra were measured on a Thermo Scientific LTQ Orbitrap Velos mass spectrometer (Thermo Fisher Scientific, Bremen, Germany). Low-resolution EI mass spectra were measured on a Thermo Electron Corporation DSQ mass spectrometer (Thermo Electron Corporation, Austin, TX, USA) using a Direct-Exposure Probe (Thermo Electron Corporation, Austin, TX, USA). Normal- and reversed-phase column chromatography separations were performed with Kieselgel Si 60 (Merck, Darmstadt, Germany) and Kieselgel RP-18 (Merck, Darmstadt, Germany), respectively. HPLC separations were conducted on a Waters 600 liquid chromatography pump (Waters, Milford, MA, USA) with a Waters 410 refractive index detector (Warers, Milford, MA, USA), using a Kromasil 100 C_18_ (250 mm × 8 mm i.d.) column (MZ-Analysentechnik GmbH, Mainz, Germany). TLC was performed with Kieselgel 60 F_254_ aluminum plates (Merck, Darmstadt, Germany) and spots were detected after spraying with 15% H_2_SO_4_ in MeOH reagent and heating at 100 °C for 1 min.

### 3.2. Biological Material

The bacterial strain BI0048 was isolated from the inner tissues of the red alga *L. glandulifera*, collected in Zoumberi bay, south of Nea Makri, Attiki, Greece in November of 2009 and was identified as *S. ambofaciens* based on comparison of its 16S rRNA sequence with data from the Genbank database of the National Center for Biotechnology Information (NCBI) using BLAST (Basic Local Alignment Search Tool) (GenBank accession number EU593561). The strain has been deposited at the strain collection/microbank of the Department of Pharmacognosy and Chemistry of Natural Products, Faculty of Pharmacy, National and Kapodistrian University of Athens.

### 3.3. Fermentation, Extraction and Isolation

The bacterial strain BI0048 was streaked from a glycerol stock onto a freshly prepared agar plate containing a seawater-based (A1BFe+C) medium (10 g starch, 4 g yeast extract, 2 g peptone, 1 g CaCO_3_, 0.1 g KBr, and 0.04 g Fe_2_(SO_4_)_3_ 5H_2_O per liter of filtered seawater) [18]. After sufficient growth of the bacterial strain was observed, mycelia were picked from the agar plate and were inoculated into 250 mL flasks containing 100 mL of the same seawater-based medium that were incubated at 37 °C for 4 days while shaking at 130 rpm in an orbit shaker. Subsequently, the starter cultures were inoculated into 2 or 3 L flasks containing 1 or 1.5 L of the same seawater-based medium (10% *v*/*v* inoculum), respectively, to a total of 21 L of liquid medium, that were incubated at 37 °C for 8 days while shaking at 130 rpm in an orbit shaker. At the end of the fermentation period, Amberlite XAD-7HP resin (Sigma-Aldrich, St. Louis, MO, USA) (20 g/L) was added to each flask to adsorb extracellular metabolites. The culture and resin were shaken overnight at low speed. The resin and cell mass were collected by filtration through cheesecloth and washed with deionized water to remove salts. The resin, cell mass and cheesecloth were then extracted for 2 h with Me_2_CO (6 L). Filtration of the extract and removal of the solvent under vacuum at 40 °C afforded a solid residue (12.5 g) that was subjected to reversed-phase vacuum column chromatography, using H_2_O with increasing amounts of MeOH, followed by MeOH with increasing amounts of CH_2_Cl_2_ as the mobile phase, to yield thirteen fractions (A–M). Fractions B, C and D were combined (0–30% MeOH in H_2_O, 2.33 g) and further fractionated by normal-phase vacuum column chromatography, using EtOAc with increasing amounts of MeOH as the mobile phase, to afford thirteen fractions (B1–B13). Fractions B1-B6 (0–25% MeOH in EtOAc, 368.9 mg) were repetitively subjected to reversed-phase HPLC, using MeOH/H_2_O (60:40) as eluant, to yield **3** (4.6 mg), **4** (5.1 mg), **5** (20.8 mg), **6** (1.3 mg), **7** (19.1 mg), **8** (1.6 mg), benzoic acid (0.7 mg), hydrocinnamic acid (13.1 mg) and (*E*)-cinnamic acid (2.4 mg). Fractions E, F and G were combined (40–70% MeOH in H_2_O, 0.93 g) and further fractionated by normal-phase gravity column chromatography, using CH_2_Cl_2_ with increasing amounts of MeOH as the mobile phase, to afford twenty-five fractions (E1–E25). Fraction E4 (5% MeOH in CH_2_Cl_2_, 122.0 mg) was further fractionated by normal-phase vacuum column chromatography, using c-Hex with increasing amounts of EtOAc as the mobile phase, to afford twelve fractions (E4a–E4l), among which fraction E4c was identified as **9** (45.8 mg). Fractions E4d (35–40% EtOAc in c-Hex, 23.1 mg) and E4e (40–50% EtOAc in c-Hex, 17.0 mg) were separately and repetitively subjected to reversed-phase HPLC, using MeOH/H_2_O (100:0 to 75:25) as eluant, to yield **1** (1.2 mg), **2** (6.6 mg), **9** (5.1 mg) and **10** (3.8 mg). Fraction E5 (5% MeOH in CH_2_Cl_2_, 134.0 mg) was further fractionated by reversed-phase vacuum column chromatography, using H_2_O with increasing amounts of MeOH as the mobile phase, to afford eight fractions (E5a–E5h). Fractions E5a and E5b were combined (30–40% MeOH in H_2_O, 54.6 mg) and purified by reversed-phase HPLC, using MeOH/H_2_O (50:50) as eluant, to yield **7** (10.6 mg). Fractions E13 and E14 were combined (13–15% MeOH in CH_2_Cl_2_, 66.0 mg) and further fractionated by reversed-phase vacuum column chromatography, using H_2_O with increasing amounts of MeOH as the mobile phase, to afford four fractions (E13a–E13d) that were repetitively subjected to reversed-phase HPLC, using MeOH/H_2_O (80:20 to 40:60) as eluant, to yield **5** (28.7 mg).

Zoumbericin A (**1**): Colorless oil; ${\left[\alpha \right]}_{D}^{20}$ +18.75 (*c* 0.05, CHCl_3_); UV (CHCl_3_) *λ*_max_ (log *ε*) 258 (4.04), 282 (3.91) nm; IR (thin film) *ν*_max_ 3246, 2918, 2850, 1687, 1561, 1460, 1250 cm^−1^; ^1^H and ^13^C NMR data, see Table 1; HRESIMS *m*/*z* 335.0928 [M − H]^−^ (calcd. for C_20_H_15_O_5_, 335.0925).

Zoumbericin B (**2**): Colorless oil; ${\left[\alpha \right]}_{D}^{20}$ +15.0 (*c* 0.09, CHCl_3_); UV (CHCl_3_) *λ*_max_ (log *ε*) 283 (3.69) nm; IR (thin film) *ν*_max_ 3307, 2918, 2850, 1688, 1561, 1456, 1249 cm^−1^; ^1^H and ^13^C NMR data, see Table 1; HRESIMS *m*/*z* 231.0660 [M − H]^−^ (calcd. for C_13_H_11_O_4_, 231.0663).

Germicidin K (**3**): Colorless oil; ${\left[\alpha \right]}_{D}^{20}$ +16.15 (*c* 0.09, CHCl_3_); UV (CHCl_3_) *λ*_max_ (log *ε*) 274 (3.59) nm; IR (thin film) *ν*_max_ 2970, 2934, 2878, 1742, 1369, 1220 cm^−1^; ^1^H and ^13^C NMR data, see Table 2; HRESIMS *m*/*z* 211.0975 [M − H]^−^ (calcd. for C_11_H_15_O_4_, 211.0976).

Germicidin L (**4**): Colorless oil; ${\left[\alpha \right]}_{D}^{20}$ +25.5 (*c* 0.07, CHCl_3_); UV (CHCl_3_) *λ*_max_ (log *ε*) 274 (3.49) nm; IR (thin film) *ν*_max_ 2971, 2918, 2851, 1737, 1367, 1218 cm^−1^; ^1^H and ^13^C NMR data, see Table 2; HRESIMS *m*/*z* 197.0818 [M − H]^−^ (calcd. for C_10_H_13_O_4_, 197.0819).

### 3.4. Evaluation of Antibacterial Activity

The antibacterial activity of compounds **1**–**10** was evaluated against the epidemic methicillin-resistant *S. aureus* strain EMRSA-15, the *S. aureus* strain SA1199B that possesses the gene encoding the NorA quinolone efflux protein and the *E. coli* strain NCTC-10418 as previously described [19].

### 3.5. Evaluation of Cytotoxic Activity

The cytotoxic activity of compounds **1**–**10** was evaluated against the MCF7 (breast adenocarcinoma) and A549 (lung carcinoma) cancer cell lines as previously described [20].

## 4. Conclusions

The chemical investigation of the organic extract of the fermentation of the endophytic strain *S. ambofaciens* BI0048, isolated from the red alga *L. glandulifera*, resulted in the isolation and structure elucidation of four new α-pyrone polyketides, namely zoumbericin A (**1**), zoumbericin B (**2**), germicidin K (**3**) and germicidin L (**4**). The evaluation of the antibacterial and cytotoxic activities of the new natural products **1**–**4** and the previously reported metabolites **5**–**10** against two multidrug-resistant strains of *S. aureus* and one strain of *E. coli*, as well as two human cancer cell lines, respectively, did not reveal any worth-nothing levels of bioactivity.

## Figures and Tables

**Table 1:** ^1^H (400 MHz) and ^13^C (50 MHz) NMR data in CDCl_3_ of compounds **1** and **2**.

Position	1		2	
	δ_C_	δ_H_ (*J* in Hz)	δ_C_	δ_H_ (*J* in Hz)
1	165.2, C ^1^	-	165.0, C ^1^	-
2	87.8, CH	5.27, d (2.2)	87.7, CH	5.39, d (2.2)
3	170.9, C	-	171.3, C	-
4	101.3, CH	5.43, d (2.2)	100.7, CH	5.68, d (2.2)
5	162.8, C	-	164.2, C	-
6	38.3, CH_2_	3.52, s	39.7, CH_2_	3.67, s
7	134.4, C ^1^	-	136.5, C	-
8	123.2, CH	6.87, d (7.5)	121.5, CH	6.78, brd (7.5)
9	133.0, CH	7.36, dd (8.2, 7.5)	130.0, CH	7.17, t (7.5)
10	116.7, CH	6.95, d (8.2)	114.5, CH	6.74, m
11	157.0, C ^1^	-	156.0, C ^1^	-
12	123.7, C ^1^	-	116.2, CH	6.73, d (1.0)
13	199.2, C ^1^	-	-	-
14	138.2, C ^1^	-	-	-
15	129.1, CH	7.67, d (8.3)	-	-
16	128.9, CH	7.43, dd (8.3, 7.4)	-	-
17	133.6, CH	7.57, t (7.4)	-	-
18	128.9, CH	7.43, dd (8.3, 7.4)	-	-
19	129.1, CH	7.67, d (8.3)	-	-
OMe	55.8, CH_3_	3.69, s	55.9, CH_3_	3.75, s
OH	-	7.80, brs	-	-

^1^ Chemical shifts were determined through HMBC correlations.

**Table 2:** ^1^H (400 MHz) and ^13^C (50 MHz) NMR data in CDCl_3_ of compounds **3** and **4**.

Position	3		4	
	δ_C_	δ_H_ (*J* in Hz)	δ_C_	δ_H_ (*J* in Hz)
1	167.2, C ^1^	-	167.6, C	-
2	91.7, C	-	91.7, C	-
3	191.2, C	-	191.3, C	-
4	104.6, CH	5.68, s	103.3, CH	5.69, s
5	175.3, C	-	176.2, C	-
6	30.7, CH_2_	1.98, q (7.6)	30.7, CH_2_	1.98, q (7.6)
7	7.3, CH_3_	0.97, t (7.6)	7.3, CH_3_	0.96, t (7.6)
8	40.2, CH	2.41, m	33.0, CH	2.64, septet (6.8)
9	26.5, CH_2_	1.68, m, 1.54, m	19.2, CH_3_	1.22, d (6.8)
10	11.4, CH_3_	0.92, t (7.4)	19.3, CH_3_	1.23, d (6.8)
11	17.1, CH_3_	1.20, d (6.9)	-	-
OH	-	9.36, brs	-	9.35, brs

^1^ Chemical shifts were determined through HMBC correlations.
